# Seaweed Extracts Enhance Salam Turfgrass Performance during Prolonged Irrigation Intervals and Saline Shock

**DOI:** 10.3389/fpls.2017.00830

**Published:** 2017-06-12

**Authors:** Hosam O. Elansary, Kowiyou Yessoufou, Amal M. E. Abdel-Hamid, Mohamed A. El-Esawi, Hayssam M. Ali, Mohamed S. Elshikh

**Affiliations:** ^1^Floriculture, Ornamental Horticulture and Garden Design Department, Faculty of Agriculture (El-Shatby), Alexandria UniversityAlexandria, Egypt; ^2^Department of Geography, Environmental Management and Energy Studies, University of JohannesburgJohannesburg, South Africa; ^3^Department of Biological and Geological Sciences, Faculty of Education, Ain Shams UniversityCairo, Egypt; ^4^Botany Department, Faculty of Science, Tanta UniversityTanta, Egypt; ^5^Botany and Microbiology Department, College of Science, King Saud UniversityRiyadh, Saudi Arabia; ^6^Timber Trees Research Department, Agriculture Research Center, Sabahia Horticulture Research Station, Horticulture Research InstituteAlexandria, Egypt

**Keywords:** *Paspalum vaginatum*, drought, salinity, seaweed extract, Salam, antioxidants

## Abstract

The negative effects of the ongoing climate change include unusual prolonged droughts and increased salinity pressures on the agricultural lands. Consequently, crops are facing unprecedented environmental pressure, and this calls for more research toward controlling such major stresses. The current study investigates the effects of seaweed extract sprays of *Ascophyllum nodosum* (5 and 7 mL·L^−1^; 6 day intervals) on *Paspalum vaginatum* Salam' during prolonged irrigation intervals (2 and 6 day) and saline growing conditions (1 and 49.7 dS·m^−1^) for 6 weeks in containers under greenhouse conditions. Control plants showed reduced turf quality, photochemical efficiency, root length and dry weight, total non-structural carbohydrates, and K and Ca compositions. Seaweed extracts increased turf quality, leaf photochemical efficiency, root length and dry weight, total non-structural carbohydrates, K, Ca, and proline in treated plants during prolonged irrigation intervals as well as saline shock conditions. There were also increases in the antioxidant defensive mechanisms such as catalase (CAT), superoxide dismutase (SOD) and ascorbate peroxidase (APX) activities and non-enzymatic antioxidants as well as reduced lipid peroxidation. The application of SWE at 7 mL·L^−1^ showed higher performance in treated plants during prolonged irrigation intervals as well as saline conditions. Our findings imply that several mechanisms including drought tolerance, osmotic adjustment and antioxidant defense system may interact to enhance the performance of plants in the face of environmental stress following SWE treatments.

## Introduction

Drought and salinity driven stress are two major agricultural challenges (Ondrasek, 2014) particularly for turfgrasses (Huang et al., 2014). In the face of the ongoing global warming and the multiplicity of environmental stresses, several investigations have been conducted to understand how turfgrasses would perform physiologically in response to drought and salinity stresses (Bian et al., 2009; Elansary and Yessoufou, 2015; Krishnan and Merewitz, 2015). In general, recent investigations on few horticultural crops indicated that seaweed extracts might be useful in enhancing the performance of few crops in drought and salinity conditions (Xu and Leskovar, 2015; Elansary et al., 2016b). Seaweed extracts (SWE) are marine algal species routinely accessible at any coastal regions around the world, and can be used for several economic purposes including soil fertilization as well as human consumption. Although, the composition of SWE is dependent on the algal species used, environmental conditions and method of preparation, the overall composition reported in many studies include polysaccharides, minerals, amino acids, and plant hormones that influence the performance of treated plants (Khan et al., 2009; Sharma et al., 2013).

*Ascophyllum nodosum* (Fucaceae) is an important species used for SWE production, and is produced commercially under several names. The SWE from *A. nodosum* and other algae have been proven to enhance horticultural crops production and performance while increasing antioxidants potential (Kumar et al., 2013; Lola-Luz et al., 2014; Elansary et al., 2016a), product quality (Kumari et al., 2011; Spann and Little, 2011), freezing tolerance (Nair et al., 2012), salinity and drought tolerance (Neily et al., 2010; Spann and Little, 2011; Guinan et al., 2013; Elansary et al., 2016b; Martynenko et al., 2016). However, the test of SWE on turfgrass performance has been rarely investigated (e.g., Zhang and Ervin, 2004; Zhang et al., 2010), although turfgrasses comprise dozens of grass species and hundreds of cultivars or accessions that are key in our daily life, e.g., home lawns, parks, golf courses, and many others (Turgeon, 2008; Hanna et al., 2013). Further, the mechanisms behind positive effects of SWE during water and saline stress conditions had not been studied before for turfgrasses. Recent investigations on SWE indicated that the method and dose of application have significant impacts on plant performance (Xu and Leskovar, 2015; Elansary et al., 2016a) also plant responses to SWE are species dependent (Spann and Little, 2011; Elansary et al., 2016b).

Plants known as seashore paspalum (*Paspalum vaginatum*) are perennial warm-season turfgrasses commonly grown worldwide in temperate, tropical and subtropical regions; they show a great diversity in texture and relatively tolerate harsh growing conditions such as salinity, drought, and water logging (Hanna et al., 2013). Newly developed cultivars of seashore paspalum tolerate low mowing heights and infrequent potable water supply, and are therefore widely grown in golf courses worldwide such as Salam, Aloha, Sea Isle 1, and Seaspray. Nonetheless, how these cultivars perform in stress conditions is poorly documented (but see Shahba et al., 2012, 2014; Elansary and Yessoufou, 2015), and particularly the potential effect of SWE on their performance is yet to be explored.

In general, turfgrasses developed three major mechanisms to resist drought. Firstly, they develop drought tolerance ability in water deficit condition, and this is achieved through osmotic adjustment driven by accumulation of solutes such as K. Secondly, they develop drought avoidance by increasing root depth to pump water and by reducing transpiration rates. The last mechanism is drought escape achieved by shortening life cycle or initiating dormancy period while in drought conditions (Nilsen and Orcutt, 1996). Due to the lack of available potable water for irrigation, there is a global trend toward the use of non-potable water, and this usually result in the accumulation of salts and the rise of salinity-driven stress for plants. Turfgrasses resist salinity conditions using physiological and biochemical mechanisms (e.g., ion homeostasis of K, fluctuation of sugar composition and production of antioxidants that detoxifying reactive oxygen species or ROS), transcriptional regulation (e.g., upregulate or downregulate specific genes such as SOS) and proteomic and metabolic regulation (e.g., production of ROS scavenging proteins including SOD and others; Mittler, 2002; Zhu, 2003; Gupta and Huang, 2014; Hussain et al., 2016). However, it is still unclear how (i.e., the mechanisms) turfgrasses respond to SWE treatments aimed to improve its physiological performance in stress conditions.

To elucidate this stress-performance relationship, the present study explores the morphological, physiological, and biochemical mechanisms involved in prolonged irrigation intervals and salinity resistance of *Paspalum vaginatum* “Salam” under *A. nodosum* SWE treatment. Studied parameters included turf quality, maximum root length and root dry weight, photochemical efficiency, total non-structural carbohydrates, K and Ca contents, and proline accumulation. The antioxidant activities by several assays were also investigated. Our rationale is that the response of turfgrasses under SWE treatment in stress conditions is driven by a complex mechanism that requires interactions among morphological, physiological, and biochemical (e.g., antioxidant) parameters.

## Materials and methods

### Plant materials

*Paspalum vaginatum* Salam sod pieces were obtained from local commercial companies in January 2016 and 2017. The plant was identified by Dr. Hosam Elansary and deposited at the Department of Floriculture, Faculty of Agriculture, Alexandria University, Egypt. Sod pieces were grown in sterilized sandy soil mixed with Crystalon® (19% N: 19% P: 19% K, Chema Industries, Egypt) at 24 kg N·ha^−1^ in PVC containers (40 cm length × 20 cm diameter) located in a greenhouse along Al-Nubariah, Alexandria-Cairo desert road. For drought stress/prolonged irrigation intervals experiment, plants were maintained for 10 h natural light conditions (23–28°C/daytime and 15–20°C/night), photosynthetically active radiation of 1000 μmol·m^−2^.s^−1^ and weekly fertilization. Plants were mowed to a 45-mm height and watered for 100% evapotranspiration rate (ET). The height of 45-mm was maintained during experiments by regular mowing. The ET was then measured on the basis of the daily change of ET in five plants through watering them and then leaving them to dry for 1 h; next, they are weighed repeatedly every 24 h and the daily weight changes, expressing the daily ET, was recorded. Volumetric water content was measured for five containers before and during the experiment through measuring the weight before and after the irrigation and by allowing free draining for 1 h. The difference between oven-dry weight (at 105°C until constant weight) and the fresh weight was recorded as the volumetric water content.

### Treatments

#### Drought experiment

Plants were subjected to two watering intervals of 2 and 6 days (2DWI and 6DWI) for 6 weeks. Watering intervals were accompanied by a 6 days treatment of *A. nodosum* SWE as a foliar spray at 5 or 7 mL·L^−1^ using a hand sprayer until drop off (leaves only). Seaweed extracts Stella Maris™ were obtained from Acadian Seaplants, Canada. Untreated plants with SWE were considered as controls. SWE treatments followed watering and started 1 week ahead of water regimes application, and this took place from the beginning to the end of the experiment (Seven SWE applications). Plants were grouped into three plots and each plot contained (at least) five replicates per treatment, totaling 90 plants (per season) distributed on three plots. Each DWI contained 15 plant per plot in each season and treatments started on January 2016 and repeated in 2017.

#### Salinity shock experiment

Plants were subjected to two salinity levels of 1 dS·m^−1^ (tap water) and 49.7 dS/m for 6 weeks. The salinity levels were obtained by gradually adding NaCl to the tank to achieve desired electrical conductivity (EC) which was measured using Metrohm™, Model 914, ON, Canada EC meter. The pH and EC levels were monitored routinely at 25°C to assure salinity levels. The salinity levels were accompanied by 6 days treatment of seaweed extracts as foliar spray at 5 or 7 mL·L^−1^ using a hand sprayer until drop off (leaves only); untreated plants were considered as controls. Nutrient solutions were supplied weekly to prevent sudden changes in growing conditions. Plants were grouped into three plots and each plot contained (at least) five replicates per treatment, totaling 90 plants (per season) distributed on three plots. Each DWI contained 15 plant per plot in each season and treatments started on January 2016 and repeated in 2017.

### Measurements

The visual quality (TQ) were measured at the end of the experiment and included plant density, uniformity and color. TQ-values were determined on a scale of 1–9, with the value 1 indicating desiccated brown turf and increasing quality, 6 acceptable quality and the value 9 is an indication of the optimum color and uniformity. The photochemical efficiency in leaves (Fv/Fm), the maximum root length (MRL), root dry weight (RDW), and the length of the deepest root were measured as described by Elansary and Yessoufou (2015). The vegetative parts were used to measure the total non-structural carbohydrates content (TNC), K^+^, Ca^2+^, and proline contents at the end of the experiment (Elansary and Salem, 2015).

The samples were freeze-dried, ground, sieved and kept in −20°C. TNC was measured for freeze-dried samples (25 mg) spectro-photometrically at a wavelength of 515 nm (Chatterton et al., 1987). K^+^ and Ca^2+^ concentrations were determined in cell sap by pressing frozen leaves (1 g); then a dilution (1:100, v/v) was used in the inductively coupled plasma spectrophotometer (Jiang and Huang, 2001). Proline composition was measured spectro-photometrically at 520 nm in 0.5 g following the methodology of Torello and Rice (1986) and Bates et al. (1973). The DPPH and β-carotene-linoleic acid assays are widely used methods in plant biochemistry for studying antioxidant activities (Pyrzynska and Pękal, 2013; Ueno et al., 2014). The DPPH assay measures the ability of compounds to scavenge free radicals or as hydrogen donor and is mixed with reducing compounds or plant materials that cause changes in the color from purple to yellow, which could be measured spectrophotometrically. β-carotene-linoleic acid is a well-known methodology for measuring antioxidant activities (Chaillou and Nazareno, 2006) and is based on the bleaching of β-carotene by the interaction with peroxyl radicals obtained during oxidation of linoleic acid. Methanolic leaf extracts were prepared to determine the antioxidant activities by 2,2′-diphenypicrylhydrazyl (DPPH), β-carotene-linoleic acid and Thiobarbituric acid (TBARS; Elansary et al., 2016b). This is done as follows: leaf methanolic extract (50 μL, 1 mg·mL^−1^) was added to 0.004% DPPH methanol solution (5 mL), and incubated in darkness for 30 min and the absorbance was then determined at 517 nm. The total antioxidant activity (TAA) represented the DPPH radical inhibition percentage. In the β-carotene-linoleic acid assay, 0.5 mg β-carotene was added to a mixture of 1 mL of chloroform, 200 mg Tween 40 and 25 μL linoleic acid then mixing and removing the chloroform by vacuum evaporation. Oxygen-saturated distilled water (100 mL) was then added to the previous mixture, followed by the addition of leaf extracts (2.5 mL mixture with 50 μL of leaf methanolic extract), and then incubated at room temperature for 48 h. The absorbance was then estimated at 470 nm and expressed in percentage (%).

A blank was prepared in the same way and antioxidant capacities of sample were compared to the blank. Thiobarbituric acid (TBARS) assay was performed using 250 μL egg yolk homogenate mixed with 50 μL of leaf methanolic extract (1 mg·mL^−1^), then completed to 500 μL using distilled water. Fe_2_SO_4_ (0.07 M, 25 μL) was added to the previous mixture and incubated at room temperature for 30 min. A mixture of 750 μL acetate buffer (20% v/v, pH 3.3), 750 μL 0.8% TBA (in SDS), and 25 μL of 20% TCA was added to the mixture and incubated at 95°C for 1 h. In addition, 1-butanol (3 mL) was added to the mixture to extract the reddish pigment, followed by centrifugation for 10 min and the upper layer was utilized to measure the absorbance at 532 nm. Blank samples were used and experiments were performed in triplicates.

Protein was extracted according to the protocol of Bradford (1976). This is briefly explained as follows: ground leaf samples were homogenized with 0.2 M phosphate buffer 1:1 (w/v) then centrifuged and the supernatant was utilized for the assay of enzyme activity. Superoxide dismutase (SOD) was measured in leaves following the manufacturer instructions using commercial kit of SOD (19160) (Sigma-Aldrich, Egypt) and the absorbance was determined at 440 nm. SOD activity represented the percentage of the inhibition of water soluble tetrazolium salt (WST-1). Catalase (CAT) and ascorbate peroxidase (APX) enzymes activities were determined in frozen leaf tissues following (Zhang and Kirkham, 1996). In brief, for CAT frozen leaf tissues (0.25 g) were homogenized on ice in 3 mL mixture of 50 mM PBS (pH 7.0), 0.2 mM EDTA and PVP (1%, w/v). The mixture was then centrifuged and the absorbance was determined at 240 nm. For APX, frozen leaf tissues (0.25 g) were homogenized in 3 mL of a mixture of PBS, M EDTA, and PVP on ice, then centrifuged and the absorbance was determined at 290 nm. To determine ROS accumulation in plants, H_2_O_2_ contents were determined in leaves using the Beyotime Colorimetric Assay Kit following manufacturer protocol (China). Free and total ascorbate were quantified by the method described by Ma et al. (2008). The mineral composition of SWE extracts was determined using Inductively Coupled Plasma Spectroscopic Analysis (ICPSA) (Optima 4300DV, Perkin-Elmer, USA). The nitrogen (N), phosphorous pentoxide (P_2_O_5_), potassium oxide (K_2_O) were determined in SWE following the AOAC methods No. 990.03, 960.08, and 960.08, respectively. Heavy metals were also quantified in SWE using AOAC method No. 6020A and experiments were repeated twice in triplicates. The sugar composition was determined by hydrolyzing dried SWE in 1 M methanolic-HCI at 80°C for 16 h. Obtained sugars were derivatized using Tri-Sil then subjected to Agilent 6890N GC-FID equipped with capillary column and hydrogen as the carrier. A temperature of 250°C was maintained for the split/splitless injection port and the detector and the injection volume was 1 μL. Different carbohydrates were expressed as percentage. To measure the expression levels of APX, CAT, DHAR, and 3 SOD isoforms genes (Cu/ZnSOD, FeSOD, MnSOD) of SWE-treated and non-treated plants grown under the saline condition of 49.7 dS.m^−1^, we used quantitative real-time PCR assay in the current study. Total RNA was isolated from these plant tissues using RNeasy Plant kit from Qiagen, followed by using RNase-Free DNase Set to remove contaminating DNA, then first strand cDNA was synthesized using Reverse Transcription kit and qRT-PCR was then performed using QuantiTect SYBR Green PCR kit (Qiagen). Gene-specific primers and PCR amplification procedures of Hu et al. (2012) and Ara et al. (2013) were used. *Actin* was used as a housekeeping gene, and 2^−ΔΔCt^ method was used for calculating the relative expression levels.

### Statistical analyses

Experimental design was used with two irrigation intervals (2 and 6DWI)/two salinity levels and three SWE treatments (5, 7 mL·L^−1^, and control), with three replications. The mean of five replicates represented the average of each treatment. The data of different irrigation intervals or salinity levels and SWE treatments of the two periods of 2016 and 2017 were analyzed using SPSS V.18 and differences among treatments were assessed using LSD at *p* = 0.05 to separate entry means in drought and salinity experiments.

## Results

### Prolonged irrigation intervals and SWE effects on turf quality, photochemical efficiency, and root parameters

Under 2 day watering intervals (2DWI), SWE treatments at 5 and 7 mL·L^−1^ had significant effects on TQ compared to control plants in both seasons (Table 1). SWE-treated plants subjected to 6 day watering intervals (6DWI) showed higher TQ-values compared to control; also TQ-values were reduced when compared to 2DWI plants. However, TQ-values were acceptable (6) in 6DWI plants treated with SWE compared to (5) in control plants. Both seasons of 2016 and 2017 showed similar pattern. In the first season and under 6DWI, leaf photochemical efficiency Fv/Fm significantly increased from 0.46 in control plants to 0.53 and 0.58 in SWE-treated plants with 5 and 7 mL·L^−1^, respectively (Table 1).

**Table 1:** Effect of irrigation intervals (2DWI and 6DWI) and SWE treatments (5, 7 ml/L, and control) on mean values of turf quality (TQ), leaf photochemical efficiency (LPE), maximum root length (MRL), root dry weight (RDW), total nonstructural carbohydrates (TNC), proline, K, and Ca of Salam turfgrass.

**Year**	**2016**						**2017**					
**Irrigation interval**	**2DWI**			**6DWI**			**2DWI**			**6DWI**		
**SWE**	**5 ml/L**	**7 ml/L**	**Control**	**5 ml/L**	**7 ml/L**	**Control**	**5 ml/L**	**7 ml/L**	**Control**	**5 ml/L**	**7 ml/L**	**Control**
TQ (1–9 scale)	8.53a^*^	8.85a	8.00b	6.00a	6.00a	5.00b	8.50a	8.75a	8.00b	7.00a	7.00a	5.00b
LPE (Fv/Fm)	0.82a	0.83a	0.80a	0.53b	0.58a	0.46c	0.82a	0.83a	0.81a	0.57b	0.61a	0.49c
MRL (cm)	30.47b	32.93a	28.11c	35.50a	36.75a	31.74b	31.62b	33.81a	28.41c	36.74a	37.95a	32.15b
RDW (mg container^−1^)	125.5b	132.26a	116.31c	136.4b	144.51a	124.32c	128.42b	135.15a	118.22c	139.11b	146.91a	126.51c
TNC (mg·g ^−1^ dry wt)	132.6a	137.2a	118.3b	101.5a	105.3a	90.1b	136.1a	142.6a	121.9b	104.3a	108.7a	93.4b
Proline (μg·g ^−1^ fresh wt)	401ab	411a	385b	1,285b	1,350a	1,218c	371ab	390a	361b	1261b	1,330a	1,191c
K (mg^.^g^−1^ dry wt)	34.0a	34.6a	32.3b	39.6a	40.9a	34.3b	34.3a	35.1a	32.7b	40.0a	41.6a	34.6b
Ca (mg^.^g^−1^ dry wt)	5.3a	5.4a	4.7b	4.4a	4.6a	3.9b	5.4a	5.5a	4.8b	4.4a	4.5a	3.9b

* *Means followed by different small letter within the same row indicate significant differences between treatments based on LSD test (P = 0.05)*.

Root parameters including maximum root length and root dry weight showed significant increases following SWE treatments compared to control plants under 2 and 6DWI in both seasons. For example, under 6DWI, the maximum root length significantly increased from 31.74 cm in control to 35.5 and 36.75 cm in 5 and 7 mL·L^−1^ SWE treated plants, respectively, in the first season. Also, maximum root length slightly increased in control plants subjected to 6DWI compared to 2DWI in both season.

### Prolonged irrigation intervals and SWE effects on TNC, proline, and K and Ca compositions

The composition of TNC showed significant increases in SWE-treated plants compared to control under 2 and 6DWI in both seasons (Table 1). The proline composition showed significant increase under 7 mL·L^−1^ SWE treatment compared to control plants under 2DWI. Also, under 6DWI both of 5 and 7 mL·L^−1^ SWE treated plants showed significantly higher proline compositions compared to control plants in both seasons.

Leaves K and Ca compositions were significantly influenced by SWE treatments in both seasons (Table 1). There were significant increases in K composition in SWE-treated plants compared to control in the first and second seasons under 2 and 6DWI. Leaves composition of Ca showed significant increases following SWE treatments in both seasons compared to control plants.

### Prolonged irrigation intervals and SWE effects on antioxidant capacity and lipid peroxidation activity

**Figure 1:**
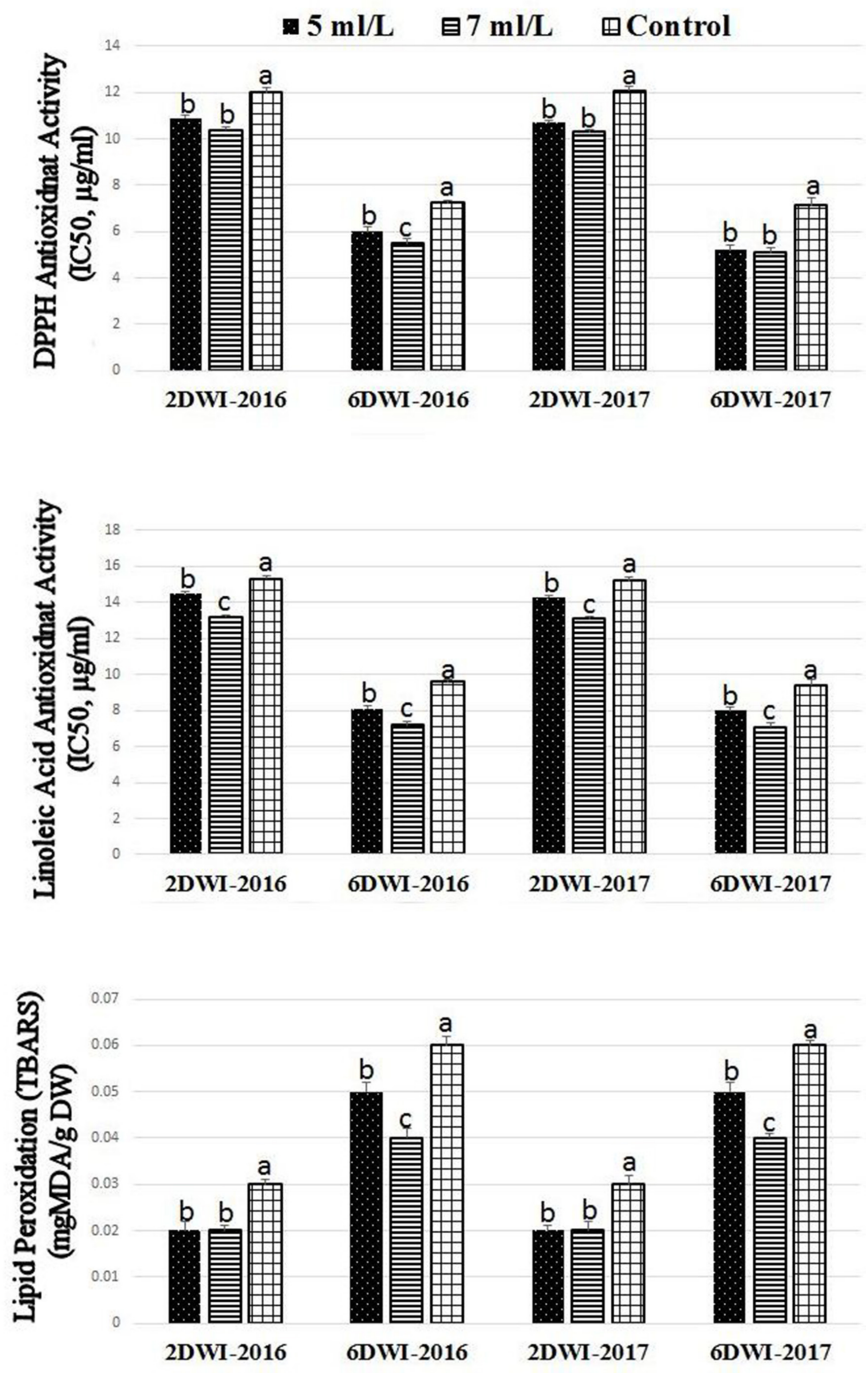
Effect of irrigation intervals (2DWI and 6DWI) and SWE treatments (5, 7 ml·L^−1^, and control) on the antioxidants activities by DPPH, β-carotene-linoleic acid, and TBARS assays in Salam turfgrass. Means followed by different small letter indicate significant differences between treatments based on LSD-test (*P* = 0.05).

SWE treatments showed significant effects on the antioxidant capacity using linoleic acid and DPPH assays as well as lipid peroxidation activity under 2 and 6DWI (Figure 1). SWE treatment significantly promoted the antioxidant activities of treated plants compared to control plants in both seasons using the linoleic acid and DPPH assays. It was noticed the higher doses of SWE treatments of 7 mL·L^−1^ showed slightly higher antioxidant activities on plants compared to 5 mL·L^−1^ in both seasons using linoleic acid assay. Activities of lipid peroxidation were lower in plants treated with SWE under 2 and 6DWI in both seasons. Also, the higher dose of SWE under 6DWI showed lower values of lipid peroxidation activities compared to the lower SWE dose. It was noted that increasing irrigation intervals from 2 to 6DWI increased the antioxidant activities in the linoleic acid and DPPH assays and reduced lipid peroxidation activity in control plants.

### Effects of salinity levels and SWE on turf quality, photochemical efficiency, and root parameters

Saline shock and SWE treatments showed significant effects on mean values of leaf photochemical efficiency, turf quality, root dry weight, and maximum root length (Table 2). Plant treated with SWE showed significantly higher turf quality than control under 1 dS·m^−1^ irrigation water in both seasons. In plants subjected to saline shock (49.7 dS·m^−1^ salinity), there were significantly higher turf quality in SWE-treated plants compared to control in both seasons. For example, in the first season, SWE treatment with 7 mL·L^−1^ had higher quality of 5.50 compared to 4.5 in control. The photochemical efficiency was higher in plants treated with SWE under saline shock (49.7 dS·m^−1^) only conditions. Under normal irrigation water (1 dS·m^−1^), SWE treatments were not effective in enhancing the photochemical efficiency in both season. The root dry weight and maximum root length were significantly higher in SWE-treated plants compared to control under both salinity levels (low and high) and in the two season.

**Table 2:** Effect of saline levels of 1 and 49.7 dS/m and SWE treatments (5, 7 ml/L, and control) on mean values turf quality (TQ), leaf photochemical efficiency (LPE), maximum root length (MRL), root dry weight (RDW), total nonstructural carbohydrates (TNC), proline, K, and Ca of Salam turfgrass.

**Year**	**2016**						**2017**					
**Salinity**	**1 dS/m**			**49.7 dS/m**			**1 dS/m**			**49.7 dS/m**		
**SWE**	**5 ml/L**	**7 ml/L**	**Control**	**5 ml/L**	**7 ml/L**	**Control**	**5 ml/L**	**7 ml/L**	**Control**	**5 ml/L**	**7 ml/L**	**Control**
Turf quality (1–9 scale)	8.51a^*^	8.83a	8.0b	5.40a	5.50a	4.50 b	8.50a^*^	8.80a	8.1b	5.50a	5.55a	4.50b
Leaf photochemical efficiency (Fv/Fm)	0.82a	0.83a	0.81a	0.64b	0.69a	0.57c	0.82a	0.83a	0.81a	0.65b	0.71a	0.58c
Maximum root length (cm)	22.21a	23.03a	20.25b	47.54a	48.33a	43.76 b	23.21a	24.11a	21.13b	49.11a	50.71a	44.76b
Root dry weight (mg container^−1^)	142.1ab	144.8a	125.7c	233.4b	247.2a	211.6c	146.1a	148.7a	124.1c	236.1b	242.6a	205.3c
Total nonstructural carbohydrates (mg·g ^−1^ dry wt)	159.2a	163.4a	148.1b	121.8a	125.6a	108.9b	156.3a	161.7a	141.1b	117.4a	121.1a	107.3b
Proline content (μg·g ^−1^ fresh wt)	385.5a	394.5a	355.0b	1,384a	1,422a	1,307b	392.1a	410.7a	340.8b	1,381a	1,451a	1,302b
K content (mg^.^g^−1^ dry wt)	33.8a	34.4a	31.9b	36.3a	36.7a	34.9b	33.4a	34.1a	31.4b	36.0a	36.7a	34.6b
Ca content (mg^.^g^−1^ dry wt)	5.2a	5.3a	4.7b	4.6a	4.6a	3.8 b	5.1a	5.2a	4.6b	4.5a	4.5a	3.9b

* *Means followed by different small letter within the same row indicate significant differences between treatments based on LSD-test (P = 0.05)*.

### Effects of saline levels and SWE on TNC, proline, and K and Ca compositions

The application of SWE during low saline as well as salinity shock significantly affected plant performance by means of TNC, proline, K and Ca compositions. The TNC mean values were significantly higher in plants treated with SWE under normal and saline shock conditions in both season. For example, TNC-values were 159.2 and 163.4 mg·g ^−1^ dry wt for SWE treated plants with 5 and 7 mL·L^−1^ compared to 148.1 mg·g ^−1^ dry wt in control plants under low salinity conditions in the first season. Proline composition was significantly increased in SWE-treated plants compared to control under low and high salinity conditions. It was also noted that proline composition increased several times in control plants subjected to saline shock compared to control in low salinity conditions. K and Ca compositions significantly increased in plants treated with SWE under low and higher salinity conditions in both season.

### Effects of saline levels and SWE on antioxidant capacity and lipid peroxidation activity

**Figure 2:**
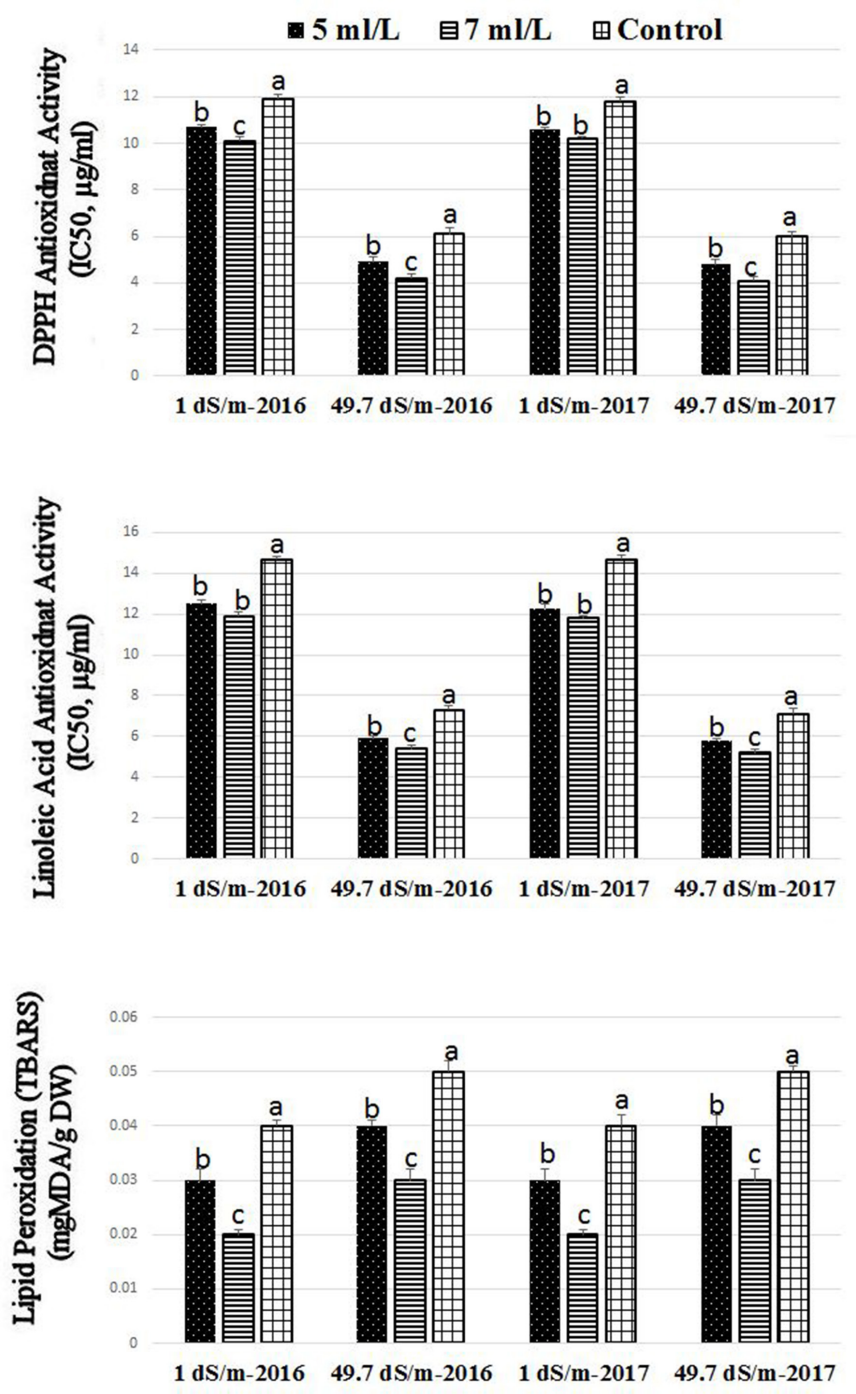
Effect of saline shock levels of 1 and 49.7 dS/m and SWE treatments (5, 7 ml·L^−1^, and control) on the antioxidants activities by DPPH, β-carotene-linoleic acid, and TBARS assays in Salam turfgrass. Means followed by different small letter indicate significant differences between treatments based on LSD-test (*P* = 0.05).

The antioxidant activities by means of DPPH and linoleic acid assay as well as lipid peroxidation activity was significantly affected by SWE treatment under low (1 dS·m^−1^) and high (49.7 dS·m^−1^) salinity conditions (Figure 2). Higher values of antioxidant activities of DPPH were found in plants treated with SWE under high and low salinity conditions in both seasons. It was also noted that SWE treatment at 7 mL·L^−1^ showed higher antioxidant activities than 5 mL·L^−1^ treatments. The linoleic acid assay showed similar pattern to that found in the DPPH; higher values of antioxidant activities were found in SWE-treated plants compared to control under low and high salinity conditions in both seasons. General increases were detected in the antioxidant activities values in the linoleic acid and DPPH assays in plants subjected to high saline compared to low saline growing conditions. Lipid peroxidation activities were significantly reduced SWE-treated plants compared to control under low and high salinity conditions.

### Effects of prolonged irrigation, saline levels and SWE on H_2_O_2_ contents, SOD, CAT, APX activities, and SWE composition

**Figure 3:**
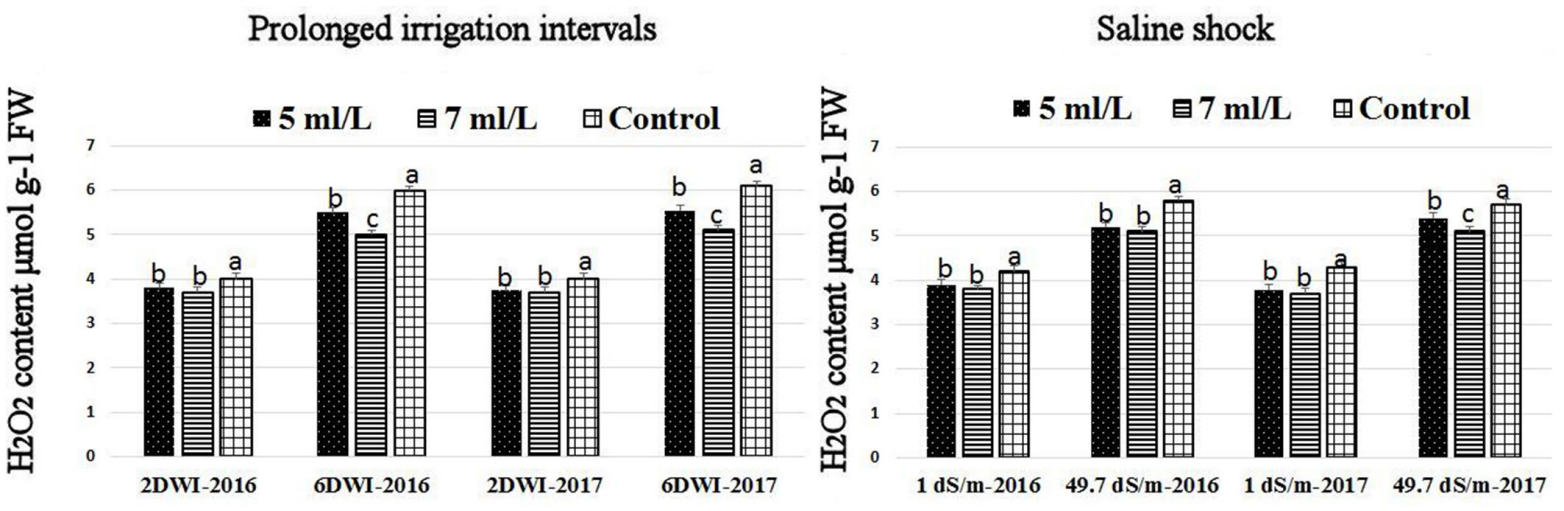
Effect of prolonged irrigation intervals **(left)** and saline shock **(right)** levels and SWE treatments (5, 7 mL·L^−1^, and control) on ROS species (H_2_O_2_) contents in Salam turfgrass leaves. Means followed by different small letter indicate significant differences between treatments based on LSD test (*P* = 0.05).

**Figure 4:**
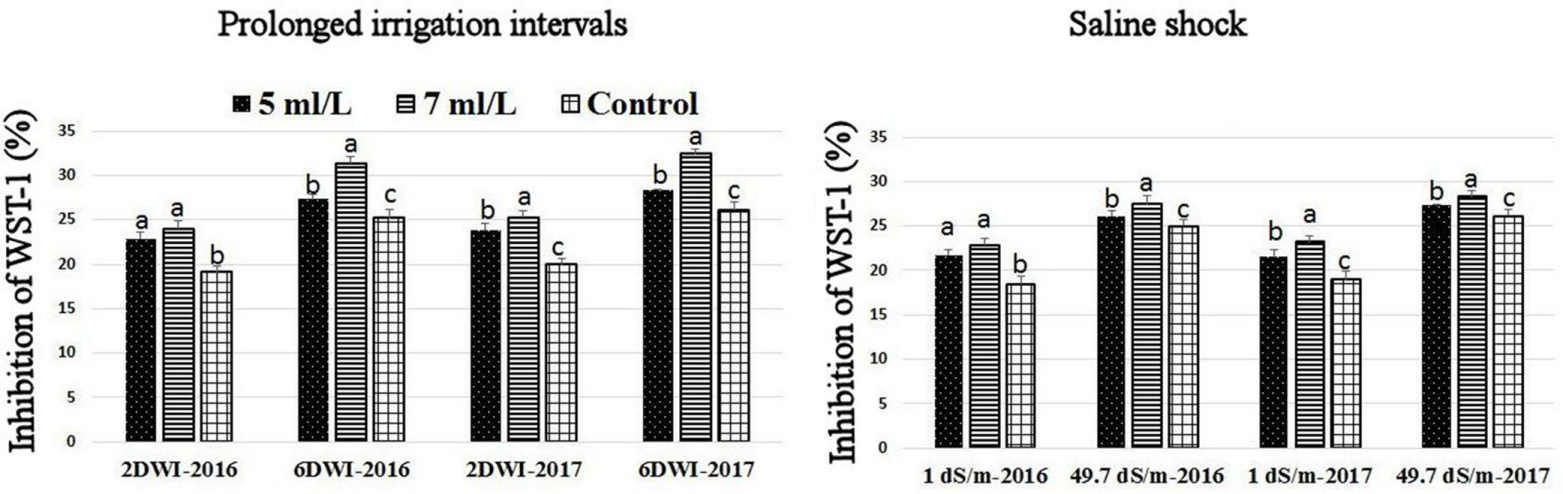
Effect of prolonged irrigation intervals **(left)** and saline shock **(right)** levels and SWE treatments (5, 7 mL·L^−1^, and control) on the inhibition of WST-1 (%) assay as an indicator of SOD enzyme activity in Salam turfgrass. Means followed by different small letter indicate significant differences between treatments based on LSD-test (*P* = 0.05).

**Figure 5:**
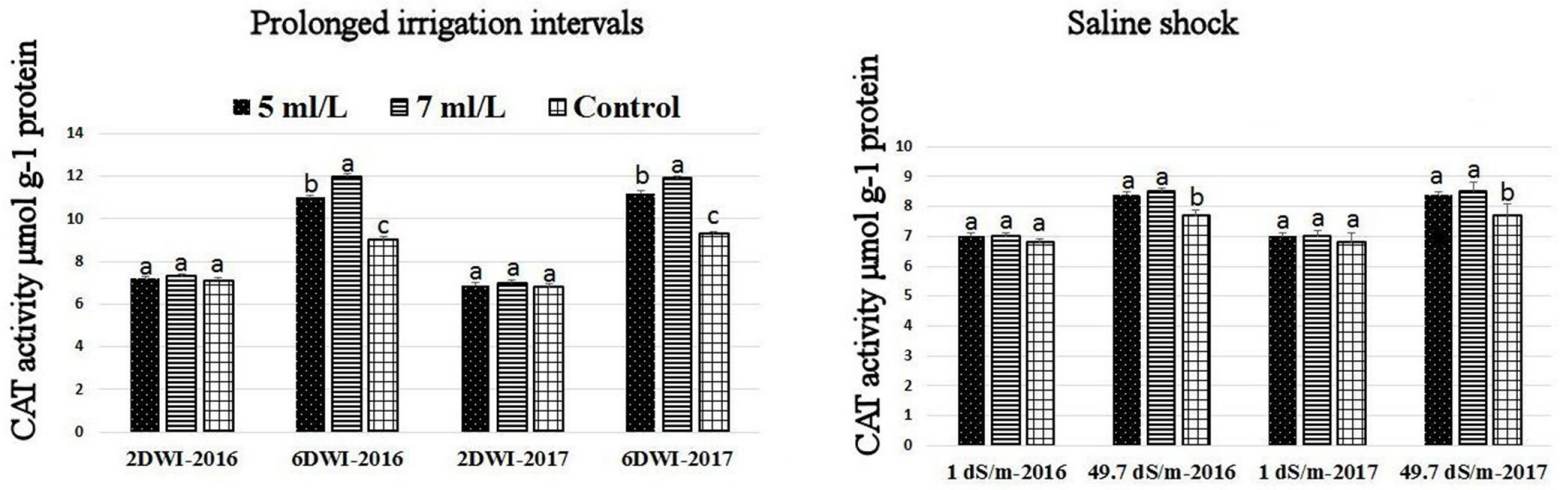
Effect of prolonged irrigation intervals **(left)** and saline shock **(right)** levels and SWE treatments (5, 7 ml·L^−1^, and control) on CAT enzyme activity in Salam turfgrass. Means followed by different small letter indicate significant differences between treatments based on LSD-test (*P* = 0.05).

**Figure 6:**
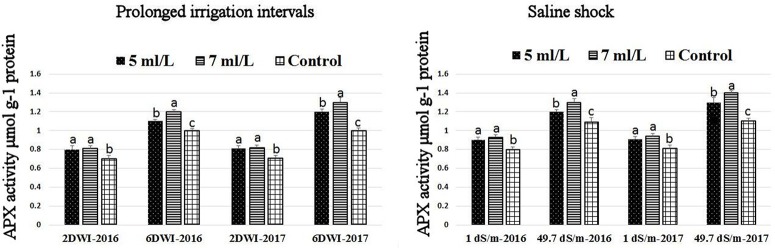
Effect of prolonged irrigation intervals **(left)** and saline shock **(right)** levels and SWE treatments (5, 7 mL·L^−1^, and control) on APX enzyme activity in Salam turfgrass. Means followed by different small letter indicate significant differences between treatments based on LSD-test (*P* = 0.05).

**Figure 7:**
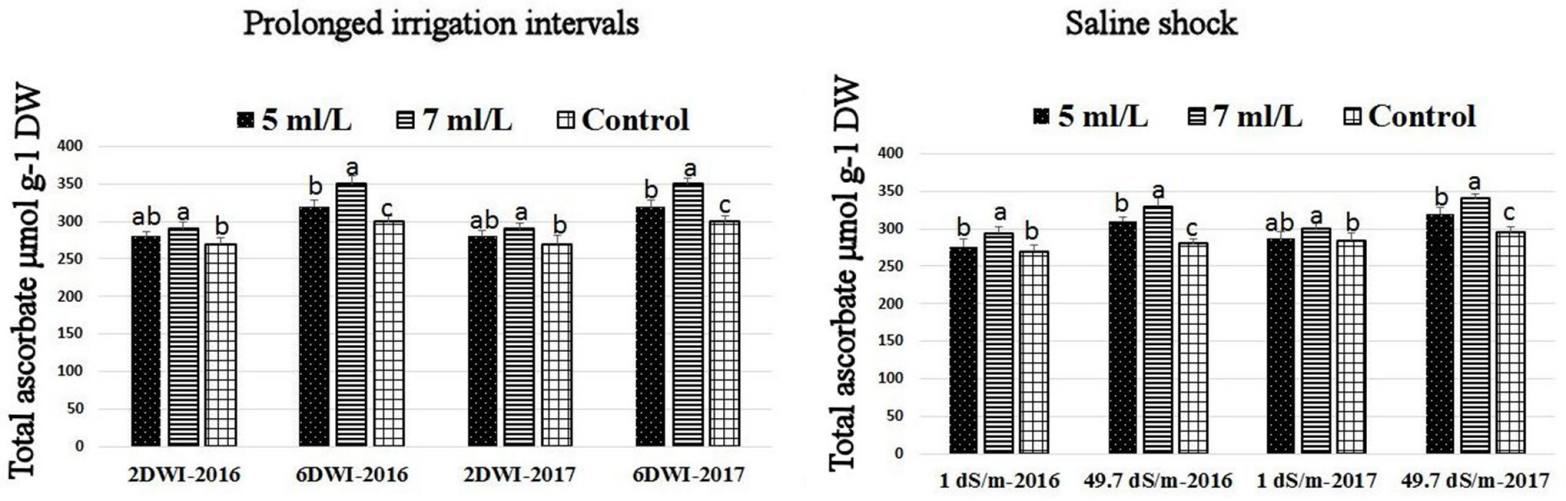
Effect of prolonged irrigation intervals **(left)** and saline shock **(right)** levels and SWE treatments (5, 7 mL·L^−1^, and control) on total ascorbate in Salam turfgrass. Means followed by different small letter indicate significant differences between treatments based on LSD-test (*P* = 0.05).

**Figure 8:**
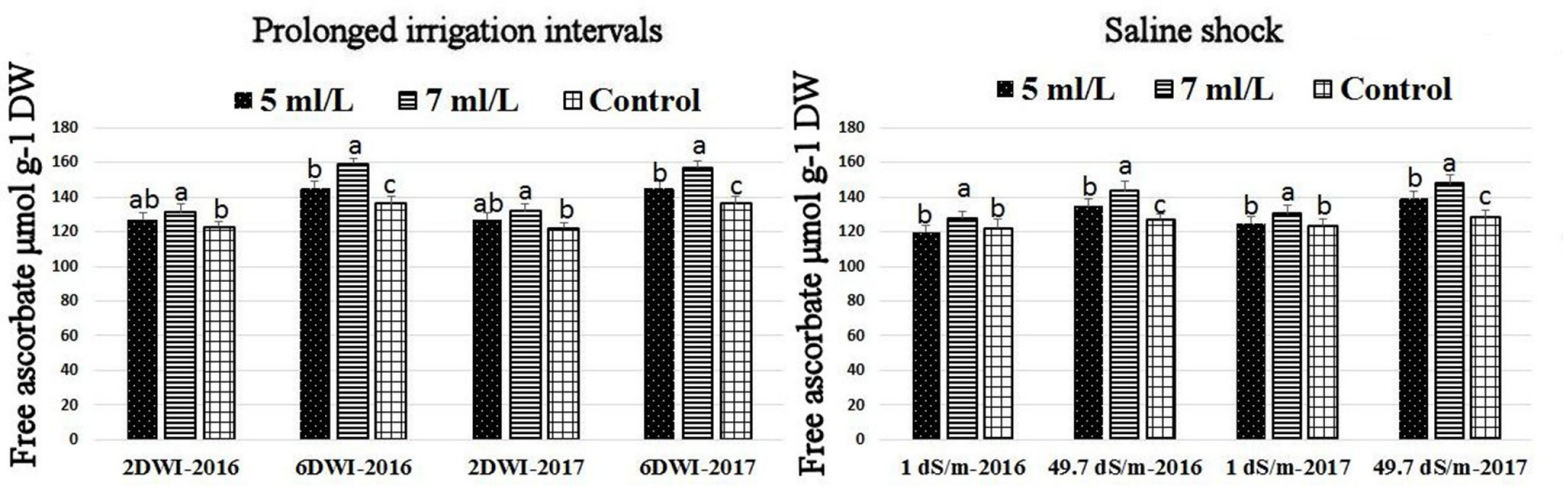
Effect of prolonged irrigation intervals **(left)** and saline shock **(right)** levels and SWE treatments (5, 7 mL·L^−1^, and control) on free ascorbate in Salam turfgrass. Means followed by different small letter indicate significant differences between treatments based on LSD-test (*P* = 0.05).

**Figure 9:**
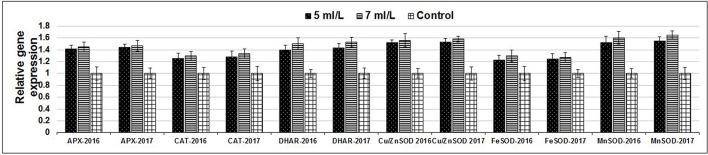
Expression levels of genes of APX, CAT, DHAR, and 3 SOD isoforms in SWE-treated and non-treated Salam turfgrass plants grown under saline condition (49.7 dS.m^−1^).

The production of ROS (H_2_O_2_) significantly increased following prolonged irrigation intervals as well as saline conditions and interestingly, SWE treatments significantly reduced ROS production in both stresses compared to untreated plants (Figure 3). There were significant increases in the inhibition of WST-1 in SWE-treated plants at 5 and 7 mL·L^−1^ compared to control under prolonged irrigation intervals as well as saline shock conditions (Figure 4). The increases in WST-1 inhibition is an indicator of the increases in SOD activity following SWE treatment. Similarly, the activities of CAT and APX enzymes significantly increased in SWE-treated plants under prolonged irrigation intervals as well as saline shock conditions (Figures 5, 6). The free and total ascorbate composition of leaves of SWE-treated plants was significantly higher than control plants (Figures 7, 8). The SWE extract used in this study contained nitrogen (N, 0.53%), phosphorus (P_2_O_5_, 0.20%), potassium (K_2_O, 0.75%), magnesium (0.1%), calcium (0.1%), trace ratios (1–5 × 10^−4^) of some important minerals such as manganese, zinc, boron, iron, and copper as well as carbohydrates 18.8% (Table 3). Major sugars found in the carbohydrates were mannitol, fucose, xylose, and glucose. Furthermore, the results of gene expression studies showed that APX, CAT, DHAR, and 3 SOD isoforms genes exhibited higher expression levels in SWE-treated plants under saline condition (49.7 dS.m^−1^), as compared to non-treated plants (Figure 9), reflecting the potential effect of seaweed extract on plants and the active role of their antioxidant enzymes under environmental and abiotic stresses.

**Table 3:** Mineral and carbohydrate compositions of SWE.

**Composition**	**%**
Nitrogen (N)	0.53 ± 0.05
Phosphate (P_2_O_5_)	0.20 ± 0.01
Soluble potash (K_2_O)	0.75 ± 0.02
Magnesium (Mg)	0.1 ± 0.02
Calcium (Ca)	0.1 ± 0.02
Sodium (Na)	0.8 ± 0.1
Iron (Fe)	5 × 10^−4^ ± 1 × 10^−5^
Copper (Cu)	3 × 10^−5^ ± 1 × 10^−6^
Zink (Zn)	1 × 10^−4^ ± 1 × 10^−5^
Manganese (Mn)	5 × 10^−5^ ± 1 × 10^−6^
Boron (B)	2 × 10^−4^ ± 1 × 10^−5^
Total carbohydrates	18.8%
Fucose	20.2 ± 0.2
Xylose	13.5 ± 0.1
Glucuronic acid	5.0 ± 0.1
Mannitol	28.5 ± 0.1
Mannose	4.9 ± 0.1
Galactose	4.9 ± 0.1
Glucose	13.06 ± 0.1
Mannuronic acid	8.2 ± 0.1

## Discussion

Water shortage as well as salinity are major challenges to the establishment of lawns and golf courses (Turgeon, 2008; Huang et al., 2014). Their effects include reduction in turf quality, photochemical efficiency and increased root depth under water stress. For example, extreme saline conditions may lead to a reduction of the photochemical efficiency in different cultivars of Paspalum Seashore (Lee et al., 2004). We found an enhancement effect of SWE on turf quality under normal (2DWI) and prolonged irrigation intervals (6DWI) as well as in plants subjected to saline shock. Such effects of SWE on turf quality had been reported in other turfgrasses subjected to heat stress such as in *Agrostis stolonifera* L. (Zhang et al., 2010) and on photochemical efficiency in *Agrostis palustris* Huds. A. subjected to cytokinin-rich SWE (e.g., Zhang and Ervin, 2004). However, these studies did not investigate different mechanisms that may interact to improve turfgrass growth during SWE treatments and prolonged irrigation or saline conditions. Such enhancement effects of SWE could be attributed to the macro- and micro-elements that made up the chemical composition of SWE as found in this study and in agreement with previous investigations (e.g., Elansary et al., 2016b). Other reports indicated the presence of cytokinins (Zhang and Ervin, 2004) and abscisic acid (Khan et al., 2009; Stirk et al., 2014) in SWE and attributed the morphological and physiological effects to such growth regulators. For example, Kumari et al. (2011) indicated that SWE may promote the photosynthetic rates by improving the chlorophyll composition of leaves, and a more recent study indicated that SWE could enhance the photosynthetic rates by reducing stomatal closure and increasing gas exchange, leading to improved growth during drought conditions (Elansary et al., 2016b).

Turfgrasses may compete water shortage by increasing root extension as found in this study in plants subjected to 6DWI, matching the well-known drought avoidance mechanism (Huang et al., 2014). The increased root depth as well as root weight in SWE treated plants subjected to prolonged irrigation intervals and saline shock conditions is in agreement with Elansary et al. (2016b) who also reported a similar pattern in ornamental shrubs following SWE application in water stress condition. Such findings prove that SWE enhances root extension as well as the photochemical efficiency in turfgrasses as drought tolerance mechanism and such conclusion had not been achieved before in turfgrasses. The optimal and effective doses of SWE for enhancing Salam turfgrass had not been investigated before, and in the current study we found that SWE application at 7 mL·L^−1^ was more effective in enhancing the photochemical efficiency and root dry weight of the plants compared to 5 mL·L^−1^. However, such effects related to dosage had been studied in other crops (Spann and Little, 2011; Mattner et al., 2013).

The accumulation of carbohydrates is a common turfgrass stress tolerance mechanism associated with osmotic adjustment during drought stress conditions (McCann and Huang, 2008; Bian et al., 2009; Elansary and Yessoufou, 2015). There were significant increases in the accumulation of TNC in SWE-treated plants under normal irrigation conditions as well as prolonged irrigation compared to control plants which might be an indication that SWE enhance the composition of TNC Salam turfgrass under normal and prolonged irrigation conditions. Such effect may lead to improved energy storage and metabolism, water and ion uptake as well as osmotic adjustment and drought resistance. SWE-TNC accumulative effect might be related to enhanced photochemical efficiency in SWE-treated plants as found in this study. Few investigations reported carbohydrates in SWE-treated plants. Goyal and Thind (2015) reported an enhanced sugar content in SWE treated rice, also Ciepiela et al. (2016) found significant increases in TNC composition in fodder grass treated with *Ecklonia maxima* SWE. Salinity leads to the significant reductions of TNC as well as increases in reducing sugars in different turfgrasses (Qian and Fu, 2005; Shahba, 2010; Shahba et al., 2012). For example, Qian and Fu (2005) reported a reduction in TNC from 123.8 to 89.4 mg·g^−1^ dry wt when salinity increased from 0.2 dS·m^−1^ (tap water) to 15 dS·m^−1^, respectively and they suggested that such reduction because TNC serves as a resource for the reduced sugars which normally increase under saline conditions. In the current study, we found parallel reductions in TNC. However, SWE had significant effects in improving the performance of treated plants in saline stress conditions.

The proline as non-protein amino acid is a known marker indicating stresses in turfgrasses such as cold, drought, and salinity and plays a pivotal role in osmoregulation of turfgrasses (Sarkar et al., 2009; Arghavani et al., 2012; Uddin et al., 2012). There were significant increases in proline values in SWE-treated plants grown under well watering conditions as well as prolonged irrigation intervals. Also, similar results were achieved in plants subjected to saline shock conditions and such results might be explained by the stimulating effects of SWE on proline accumulation. Proline response to SWE application had been reported before in few plants including *Agrostis stolonifera* var. “Penn A4” (Butler and Hunter, 2007) and *Spiraea nipponica* “Snowmound” and *Pittosporum eugenioides* “Variegatum” (Elansary et al., 2016b). They described suppressive effects of SWE on proline accumulation. However, Munshaw et al. (2006) reported that SWE have no consistent effect on proline composition in *Cynodon* spp. cultivars. Shahba et al. (2012) suggested an important role of proline in salinity tolerance in seashore paspalum cultivars. Proline accumulation in plants was described as osmotic strength enhancer and stimulate plant free radical scavenging (Gill and Tuteja, 2010; Rai et al., 2012) and we found positive effects of proline accumulation in this study in response to SWE sprays on Salam turfgrass. Further, Lee et al. (2008) reported that increased proline accumulation in other paspalum seashore cultivars is directly related to salinity tolerance in specific genotypes.

The accumulation of specific ions such as K and Ca in the vegetative parts of plants had been associated with salinity and drought conditions as a mechanism for osmotic adjustment. In turfgrasses, the accumulation of ions such as K and Ca was associated with TNC build up in stressed plants (Lu et al., 2007; Gurmani et al., 2011). This accumulation of these ions plays an important role in keeping cell turgor during stress conditions and modulates Na homeostasis (Gupta and Huang, 2014), especially K, which plays a critical role in stress response in plants Wang et al. (2013) There were significant increases in K and Ca composition in the leaves of SWE-treated plants compared to control under prolonged irrigation intervals as well as saline conditions. Such effect might be attributed not only to the chemical composition of SWE, which include important minerals such as K, but also to the enhanced photosynthetic rates driven by SWE treatement, leading to TNC accumulation and improved ion transport. The positive effects of SWE on mineral composition of treated plants had been reported in several plants, e.g., *Lepidium sativum* (Godlewska et al., 2016) and others (Arioli et al., 2015) which is in agreement with the current study.

Under stress conditions plants tend to produce reactive oxygen species (ROS) that include superoxide, hydroxyl, perhydroxy, and alkoxy radicals and these molecules may attack and damage cellular compartments such as DNA, thus producing proteins and membranes unsaturated lipids (Gill and Tuteja, 2010; AbdElgawad et al., 2016). Plants developed mechanisms that control ROS production during stress conditions by increased production of antioxidants as well as the activation of antioxidant enzymes as defense system. Some turfgrasses tend to increase their composition in unsaturated lipids to survive stresses (Huang et al., 2014) and this production has been associated with increased lipid peroxidation in stress conditions (Bian and Jian, 2009; Liu et al., 2012). So far, only few studies investigated the effects of SWE on the antioxidant activity on turfgrasses, e.g., Zhang and Schmidt (2000) reported that SWE might enhance the antioxidant activities of *Agrostis palustris* Huds. A. by means of increased composition of α-tocopherol and ascorbic acid. However, our study is the first to investigate the effects of SWE application on drought and salinity tolerance of Paspalum Seashore cultivar. In addition, few studies indicated that SWE application might enhance the antioxidant activities by means of increased flavonoid and phenolic composition (Fan et al., 2011; Lola-Luz et al., 2014; Elansary et al., 2016a). In this study, significant increases in the overall antioxidant activities in plants treated with SWE during drought and saline conditions were recorded. Furthermore, we found significant increases in SOD, CAT, and APX enzymes activities following SWE applications compared to control plants under prolonged irrigation intervals as well as saline shock conditions. The expression levels of APX, CAT, DHAR, and 3 SOD isoforms genes revealed higher increase in SWE-treated plants under saline condition (49.7 dS.m^−1^), as compared to non-treated plants, confirming the potential effect of seaweed extract on plants and the active role of their antioxidant enzymes under environmental and abiotic stresses. Enzymes such as CAT, SOD, and APX reduce ROS (H_2_O_2_) to water; we also found that ascorbate as non-enzymatic antioxidant plays an important role in the overall reduction of ROS accumulation. Such increases in the enzymatic and non-enzymatic reducing compounds are an indication that SWE treatment in Salam turfgrass mitigates stress conditions by stimulating the antioxidant mechanism pathway. SOD enzyme catalyzes superoxide radicals to form oxygen and hydrogen peroxide. The increases in SOD activities in leaves is associated with drought (Fu and Huang, 2001) and salinity (Hussain et al., 2016). Recent investigation by Zong et al. (2015) reported increased ROS scavenging enzymes activities such as SOD and peroxidase (POD) in *Paspalum vaginatum* following stress conditions (e.g., water logging). Also Xu et al. (2015) found increased production of ROS (O_2_ and H_2_O_2_) in the root of *Festuca arundinacea* Schreb. in plants subjected to water stress compared to control and this increase accompanies increases in the activities of CAT and APX enzymes in the root maturation zone. However, root elongation zone did not show similar pattern. Zang et al. (2017) found that heat stress in *Triticum aestivum* L. increased ROS production (H_2_O_2_). In agreement with our results, Chen et al. (2016) found that salinity and tolerance genes in *Paspalum vaginatum* are associated with the antioxidant system and the photosynthetic metabolism. Also, a high dose of SWE (7 mL·L^−1^) was more effective in increasing the antioxidant activities as well as the activities of specific enzymes and reducing the lipid peroxidation values and such dose dependent variation had been reported elsewhere in few crops (Spann and Little, 2011; Mattner et al., 2013; Elansary et al., 2016a). Sugars as found in the SWE used in this study had been associated with signaling pathways of plant hormones, the accumulation of auxins and works as elicitors that accumulate secondary metabolites in plants (Rayorath et al., 2008a,b; Vera et al., 2012) which adds more complexity to the mechanisms driving SWE stress tolerance stimulated effect.

In the current study several mechanisms (e.g., drought tolerance, osmotic adjustment, and antioxidant defense system) were found to influence SWE-treated turfgrass performance during stress conditions. Indeed, other mechanisms may influence such performance including polysaccharide-driven natural defense responses (Khan et al., 2009), hormonal upregulation (Zhang and Ervin, 2004), and betaines (MacKinnon et al., 2010) which require several studies to understand such complex activity. However, from the current study, we have a strong ground to believe that SWE may mitigate side effects of prolonged irrigation intervals due to competitiveness on water resources as well as saline shock conditions when using high saline waters to overcome lack of potable water during drought periods known to occur in most agricultural areas around the world.

## Conclusion

This is the first study investigating different mechanisms driving positive effects of SWE during water and saline stress conditions in turfgrasses. Environmental stresses such as prolonged irrigation intervals or deficit irrigation and salinity may reduce turf quality and photosynthetic rate; increase root length and dry weight; diminish TNC, K and Ca compositions; inhibit antioxidant defense systems and increase lipid peroxidation. Our findings provide evidence that the application of *A. nodosum* SWE may mitigate drought and salinity effects and enhance the overall performance of Salam turf grass by increasing turf quality, photosynthetic rate, root length and dry weight (drought tolerance), TNC, K and Ca leaf composition (osmotic adjustment), overall antioxidant activity, SOD, CAT and APX activities, and inhibiting lipid peroxidation (antioxidant defense system).

## Author contributions

HE and KY designed the experiment, HE, AA, and ME performed experiments, all authors contributed in validating, writing, and approving the final version of the manuscript.

### Conflict of interest statement

The authors declare that the research was conducted in the absence of any commercial or financial relationships that could be construed as a potential conflict of interest.
